# Microbial community structure and function on sinking particles in the North Pacific Subtropical Gyre

**DOI:** 10.3389/fmicb.2015.00469

**Published:** 2015-05-19

**Authors:** Kristina M. Fontanez, John M. Eppley, Ty J. Samo, David M. Karl, Edward F. DeLong

**Affiliations:** ^1^Department of Civil and Environmental Engineering, Massachusetts Institute of TechnologyCambridge, MA, USA; ^2^Department of Oceanography, School of Ocean and Earth Science and Technology, University of HawaiiHonolulu, HI, USA; ^3^Daniel K. Inouye Center for Microbial Oceanography: Research and Education, University of HawaiiHonolulu, HI, USA; ^4^Lawrence Livermore National Laboratory, Nuclear and Chemical Sciences DivisionLivermore, CA, USA

**Keywords:** metagenomics, marine particles, sediment trap, biological pump, microbiology

## Abstract

Sinking particles mediate the transport of carbon and energy to the deep-sea, yet the specific microbes associated with sedimenting particles in the ocean's interior remain largely uncharacterized. In this study, we used particle interceptor traps (PITs) to assess the nature of particle-associated microbial communities collected at a variety of depths in the North Pacific Subtropical Gyre. Comparative metagenomics was used to assess differences in microbial taxa and functional gene repertoires in PITs containing a preservative (poisoned traps) compared to preservative-free traps where growth was allowed to continue *in situ* (live traps). Live trap microbial communities shared taxonomic and functional similarities with bacteria previously reported to be enriched in dissolved organic matter (DOM) microcosms (e.g., *Alteromonas* and *Methylophaga*), in addition to other particle and eukaryote-associated bacteria (e.g., *Flavobacteriales* and *Pseudoalteromonas*). Poisoned trap microbial assemblages were enriched in *Vibrio* and *Campylobacterales* likely associated with eukaryotic surfaces and intestinal tracts as symbionts, pathogens, or saprophytes. The functional gene content of microbial assemblages in poisoned traps included a variety of genes involved in virulence, anaerobic metabolism, attachment to chitinaceaous surfaces, and chitin degradation. The presence of chitinaceaous surfaces was also accompanied by the co-existence of bacteria which encoded the capacity to attach to, transport and metabolize chitin and its derivatives. Distinctly different microbial assemblages predominated in live traps, which were largely represented by copiotrophs and eukaryote-associated bacterial communities. Predominant sediment trap-assocaited eukaryotic phyla included *Dinoflagellata, Metazoa* (mostly copepods), *Protalveolata, Retaria*, and *Stramenopiles*. These data indicate the central role of eukaryotic taxa in structuring sinking particle microbial assemblages, as well as the rapid responses of indigenous microbial species in the degradation of marine particulate organic matter (POM) *in situ* in the ocean's interior.

## Introduction

Particulate organic matter (POM) generated in the euphotic zone is the major conduit of matter and energy transport to the deep sea and also represents the primary mechanism of carbon removal from surface waters via the biological pump (McCave, 1975; Volk and Hoffert, 1985). POM is operationally defined as particles ranging from 0.1 μm to centimeters in size, and is further qualitatively subcategorized into macroaggregates (marine snow; centimeters to 500 μm in diameter), microaggregates (500–1 μm), and submicron particles (1–0.1 μm) (Simon et al., 2002). Sinking POM can be collected *in situ* using sediment traps that contain saline solutions slightly denser than seawater that retain sinking particles (Knauer et al., 1979). This broad size spectrum of POM harbors a diverse and complex variety of inorganic as well as living and non-living organic materials (Volkman and Tanoue, 2002; Nebbioso and Piccolo, 2013).

Much of the current knowledge of POM-degrading microbial communities is derived from studies of suspended POM. Analysis of whole seawater segregated into particle-associated (>1 μm) and free-living size fractions has revealed taxonomically and functionally distinct microbial communities in marine anoxic zones (Ganesh et al., 2014), coastal ecosystems (Allen et al., 2012; Smith et al., 2013), estuarine environments (Crump et al., 1999; Waidner and Kirchman, 2007), inland seas (Moeseneder et al., 2001; Fuchsman et al., 2011, 2012; Crespo et al., 2013), phytoplankton blooms (Riemann et al., 2000; Fandino et al., 2005; Teeling et al., 2012), ocean trenches (Eloe et al., 2011), and the open ocean (Kellogg and Deming, 2009; Allen et al., 2012). These studies have shown that in particular, members of the *Bacteroidetes, Planctomycetes*, and *Deltaproteobacteria* are often enriched in larger particle size fractions. Studies of microbial community composition on sinking particles are less extensive than those on suspended particles. Research programs such as the Vertical Transport and Exchange (VERTEX)(Martin et al., 1987) and VERtical Transport In the Global Ocean (VERTIGO) (Buesseler et al., 2008) supported diverse process-oriented studies that revealed the importance of chemolithotrophs like nitrifiers (Karl et al., 1984), organotrophs (Boyd et al., 1999), and exoenzyme-driven degradation on sinking particles (Smith et al., 1992). These findings laid the foundation for phylogentically-oriented studies that suggested that *Bacteroidetes, Planctomycetes*, and *Roseobacter* can act as sinking particle colonizers in the upper water column (DeLong et al., 1993; LeCleir et al., 2013). While sediment traps have proven useful for over 30 years in studies of sinking POM (Karl and Knauer, 1984), to date there exists only one report of the phylogenetic diversity of sediment-trap collected microbes, which grew over 24 h in sediment-trap captured particles from 100 to 120 m (LeCleir et al., 2013).

Given the diverse sources and sinks of sinking particles in the ocean's interior (Honjo et al., 2008), much remains to be learned about the microbes and processes that regulate the degradation of sinking POM. In this study, we sought to examine the nature of sinking particles collected in poisoned traps, which we hypothesized would help preserve sinking materials and allow us to identify (using metagenomics) the sources of larger sinking particulates including larger eukaryotes that are known to aggregate and sink to the deep-sea. We also included paired, un-poisoned traps (live) in our experiments, postulating that these might reveal the nature and identity of microorganisms capable of growth on the collected organic material at the *in situ* temperatures and pressures of trap deployment. We reasoned that the phylogenetic identity of poisoned vs. live traps would reveal the identity and genomic potential of microbes capable of growth on sinking particulate organic materials *in situ* in the ocean's interior.

## Materials and methods

### Sample collection

A free-drifting sediment trap array identical to those used in the VERTEX and HOT field programs (Knauer et al., 1979) was deployed at station ALOHA (22.75°N, 158°W) in the North Pacific Subtropical Gyre on July 14, 2012. Each trap tube (cross sectional area of 0.0039 m^2^) was filled with approximately 1.8 liters of either an 0.2 μm-filtered brine solution (Knauer et al., 1984) (“live”) or an 0.2 μm-filtered RNAlater solution (“poisoned”) adjusted to a density of 1.05 g/cc (see Supplementary Material, for further methodological details on trap solutions). Both sets of traps were fitted with a 335 um Nitex screen below the topmost baffle in order to exclude larger zooplankton. The array drifted north-west for 75 nautical miles before recovery on July 26, 2012. Prior to filtration, the 335 μm Nitex screen was removed along with approximately 500 mL of seawater overlying the higher density hypersaline trap solution. Following recovery, particles in the 0.2–335 μm fraction were collected on Sterivex filters (EMD Millipore, Billerica, MA, USA) and preserved with 1.5 mL RNAlater (Ambion, Carlsbad, CA, USA). Filters were stored at −80°C prior to nucleic acid isolation.

### Microscopy

#### Epifluorescence and optical microscopy

For each depth, 10 mL of fixed sediment trap sample (2% formaldehyde final concentration) was filtered onto black 0.2 μm pore size polycarbonate filters and allowed to dry completely. Ethanol cleansed surgical scissors were used to cut 1/8 pieces from each filter and four pieces were then positioned on a microscope slide (Fisherbrand Superfrost precleaned microscope slides #12-550-143). A 24 × 50 mm coverslip with #1.5 thickness was placed below the slide. Antifade mounting medium (Patel et al., 2007) containing 1 μg mL^−1^ of nucleic acid stain 4′,6-diamidino-2-phenylindole (DAPI) was spotted on the coverslip (15 μL) to align with the filter pieces. The coverslip was inverted onto the slide and the filters were stained for 10 min. Filters were visualized at 1000× total magnification on a Nikon 90i epifluorescence microscope with excitation/emission settings for DAPI, chlorophyll, and phycoerythrin. Images were acquired with a QImaging Retiga EXi camera using optimized exposure times and analyzed with Nikon NIS-Elements software.

For optical microscopy, unmounted filters were visualized through a Nikon AZ100 Multizoom microscope with a 10× objective at 20× and 40× magnification (zoom settings 4 and 8) using a Nikon NI-150 illuminator. Images were captured via NIS-Elements software using a Nikon DS-Fi1 camera.

### Library preparation and sequencing

Approximately one-half of each Sterivex filter was used for extraction of total community DNA using the Powerwater DNA isolation kit (Mobio, Carlsbad, CA, USA), with modifications (see Supplementary Material for details). Library preparation followed the Nextera XT DNA sample preparation protocol (Illumina, San Diego, CA, USA). Samples were dual indexed and 10 samples pooled per sequencing run on a MiSeq using MiSeq reagent kit v3 (Illumina, San Diego, CA, USA). Sequencing and quality control followed the manufacturer's recommendations.

### Sequence analysis and annotation

Sequencing and annotation statistics for sediment trap and seawater samples are summarized in Table A1 in Supplementary Material. Metagenomic sequences were filtered with Trimmomatic (Bolger et al., 2014), PandaSeq (Masella et al., 2012), and SortMeRNA to identify rRNA-containing reads (Kopylova et al., 2012) as previously described (Lincoln et al., 2014) with one modification. Unjoined read pairs output from PandaSeq were joined with 6 N's and tracked along with paired reads. The taxonomic origins of rRNA and non-rRNA reads were determined by comparison against SILVA release 115 and the NCBI RefSeq release 61 databases, respectively, using lastal (Kiełbasa et al., 2011). Function of non-rRNA reads was determined by comparison with the September 2013 version of the Kyoto Encyclopedia of Genes and Genomes (KEGG) (Kanehisa and Goto, 2000) database using lastal and the March 22, 2013 version of the Carbohydrate Active Enzyme (CAZy) database (Cantarel et al., 2009) using HMMER3.0 (Eddy, 2011) and hidden Markov models of CAZy signature domains (Yin et al., 2012). See Supplementary Material for further details.

### Statistical analyses

**Figure 1:**
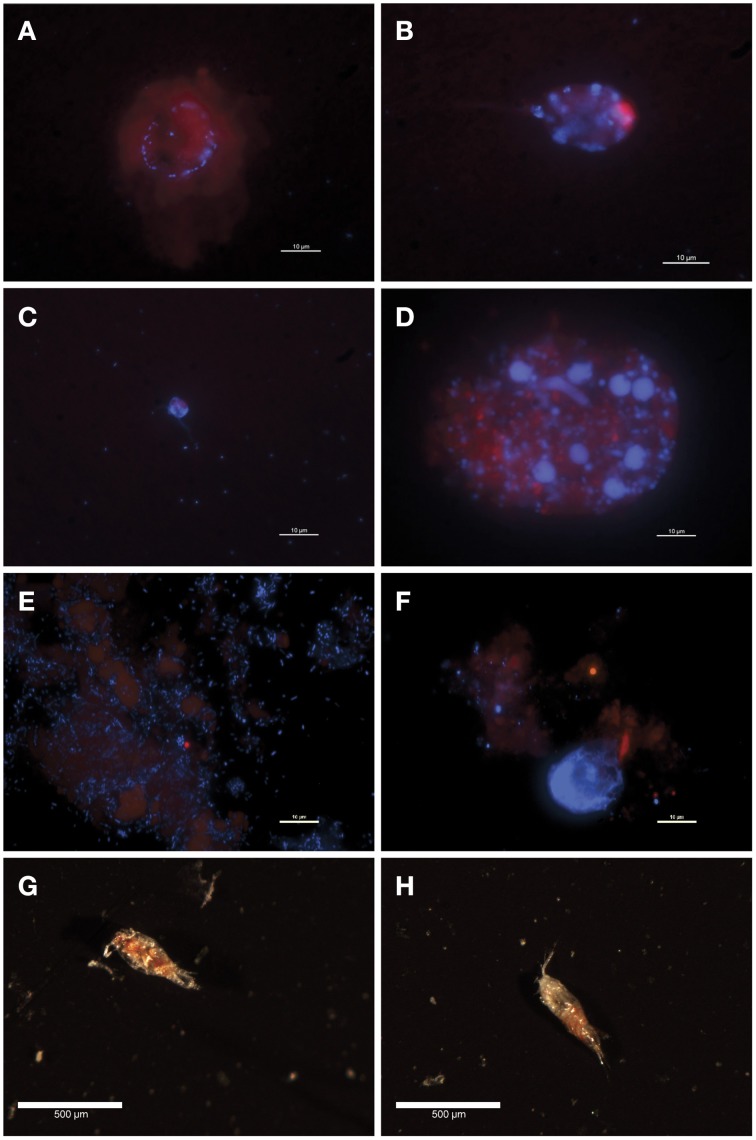
**Fluorescence (A–F) and optical (G,H) microscopy images of sediment trap POM. (A)** 150m, poisoned trap; A circle of bacteria associated with a chlorophyll-containing particle **(B)** 200 m, poisoned trap; Unattached, ovoid pigmented cell in class Dinophyceae **(C)** 300 m, poisoned trap; A dually flagellated cell in class Chrysophyceae loosely surrounded by bacteria **(D)** 500 m, poisoned trap; A chlorophyll-containing particle covered with non-pigmented cells of varying sizes including flagellates in class Chrysophyceae and large bacteria **(E)** 150 m, live trap; Abundant bacteria, some associated with diffuse chlorophyll-containing particles **(F)** 150 m, poisoned trap; Sparsely distributed bacteria associated with a chlorophyll-containing particle **(G)** 150 m, live trap; Partially degraded copepod **(H)** 150 m, poisoned trap; Slightly degraded copepod.

All metagenomic sequence counts were normalized and variance stabilized using the regularized log transformation in DESeq2 (Love et al., 2014). Ordination of normalized sequences used principal coordinate analysis with Bray-Curtis distance in the phyloseq R package (McMurdie and Holmes, 2013). Significance (*p* < 0.05) of clusters was determined using non-parametric analysis of variance based on dissimilarities in the vegan R package (Dixon, 2003). A negative binomial Wald test in DESeq2 was used to identify statistically significant differences in taxonomic and functional non-normalized gene counts among live traps, poisoned traps, and seawater (data not shown). The presence of copepods in live and poisoned traps was also confirmed by optical microscopy (Figures 1G,H). As replicates of sediment traps at each depth were not available, all four depths belonging to a treatment were modeled as biological replicates. A false discovery rate threshold of 0.01 was used for detecting differentially abundant taxa or functions. For statistical validation of depth-specific taxonomic differences, Fisher's exact test as implemented in the STAMP v2.01 program (Parks and Beiko, 2010) was used for pairwise comparisons of 150 m vs. 500 m RefSeq-identified non-normalized taxa within treatment types. A false discovery rate threshold of 0.05 and a difference between proportions cutoff of 1 were used to assess statistical and biological significance, respectively.

### Sequence data

The sequences reported in this paper have been deposited in the Genbank Short Read Archive (Bioproject PRJNA270248).

## Results and discussion

Sinking particles were collected using a free-drifting sediment trap array deployed in the North Pacific Subtropical Gyre, from the base of the photic zone and into the mesopelagic [150, 200, 300, and 500 m (Figure A1 in Supplementary Material)]. The sediment traps included two treatments: a “live” trap which contained a solution of sterile seawater adjusted to a density of 1.05 g/cc with NaCl and a “poisoned” trap which contained a preservative to prevent *in situ* growth and preserve DNA. Fluorescence microscopy of trap-collected POM revealed bacteria associated with chlorophyll-containing particles in both live and poisoned sediment traps (Figures 1A,D–F). Bacteria in the sediment traps were either unattached, loosely surrounding protists (Figure 1C), or directly associating with amorphous particles (Figure 1D). Unattached bacteria were most abundant in 150 m live traps likely due to the opportunity for enhanced growth during deployment (Figure 1E).

### Domain level taxonomic composition in live vs. poisoned sediment traps

**Figure 2:**
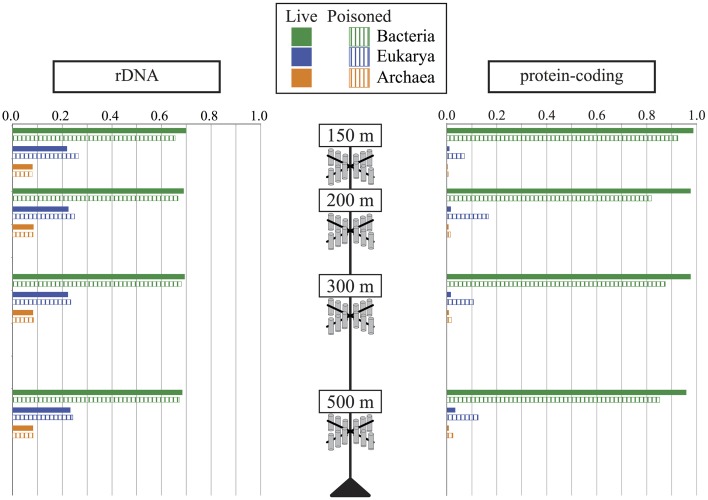
**The relative abundance of Archaea, Bacteria, and Eukarya in live (filled bars) and poisoned (striped bars) sediment traps at the indicated depths as determined by the taxonomic identifications of small subunit ribosomal RNA genes (rDNA) and protein coding genes**.

DNA was extracted and shotgun sequenced from both live and poisoned sediment trap particulates collected at the base of the photic zone and into the mesopelagic (Figure A1 in Supplementary Material) to determine the taxonomic and functional diversity associated with sinking particles. The diversity of the particle-associated microbes at the domain-level indicated the dominance of Bacteria and Eukarya in all traps at all depths sampled (Figure 2). Archaea represented less than 10% of total in both live and poisoned sediment trap microbial assemblages at all depths (Table 1).

**Table 1:** **The relative abundance of Bacteria, Eukaryota, and Archaea in live (L) and dead (D) sediment traps**.

	**rDNA^a^**							
	**150L^b^**(%)	**150D (%)**	**200L (%)**	**200D (%)**	**300L (%)**	**300D (%)**	**500L (%)**	**500D (%)**
Bacteria	70.0	65.6	69.1	66.7	69.4	68.1	68.4	67.3
Eukaryota	22.0	26.6	22.5	25.0	22.3	23.5	23.3	24.3
Archaea	8.0	7.8	8.4	8.2	8.3	8.5	8.3	8.3
	**Protein-coding^c^**							
	**150L (%)**	**150D (%)**	**200L (%)**	**200D (%)**	**300L (%)**	**300D (%)**	**500L (%)**	**500D (%)**
Bacteria	98.7	92.4	97.6	81.9	97.6	87.4	95.9	85.1
Eukaryota	1.0	7.0	1.6	16.6	1.7	10.7	3.4	12.5
Archaea	0.3	0.6	0.7	1.5	0.8	1.9	0.8	2.4

a *Normalized relative abundance determined by rDNA, small subunit ribosomal RNA genes*.

b *Numbers preceding L and D refer to sediment trap depths in meters (150, 200, 300, and 500 m)*.

c *Normalized relative abundance determined by protein-coding genes*.

Eukaryotic rRNA genes were more highly represented than identifiable protein-encoding sequence reads, likely due to the inherently lower gene density in eukaryotes vs. bacteria, as well as the lack of closely related eukaryotic reference genomes for a number of highly represented taxa. Notably, the percentage of total annotated genes in poisoned traps was consistently lower than that of live traps (Table A1 in Supplementary Material). This result most likely reflects the higher relative abundance of eukaryotic DNA in the poisoned traps compared to live traps. In total, these results are consistent with previous observations that particles are more enriched in eukaryote-associated and unclassified protein-coding genes (Allen et al., 2013; Smith et al., 2013; Ganesh et al., 2014). The decreased ratio of eukaryote-to-bacteria DNA in the live traps was apparently due to *in situ* microbial growth in the trap during the deployment as POM underwent decomposition, and possibly concomitant degradation of the particle-associated eukaryotic nucleic acids.

**Figure 3:**
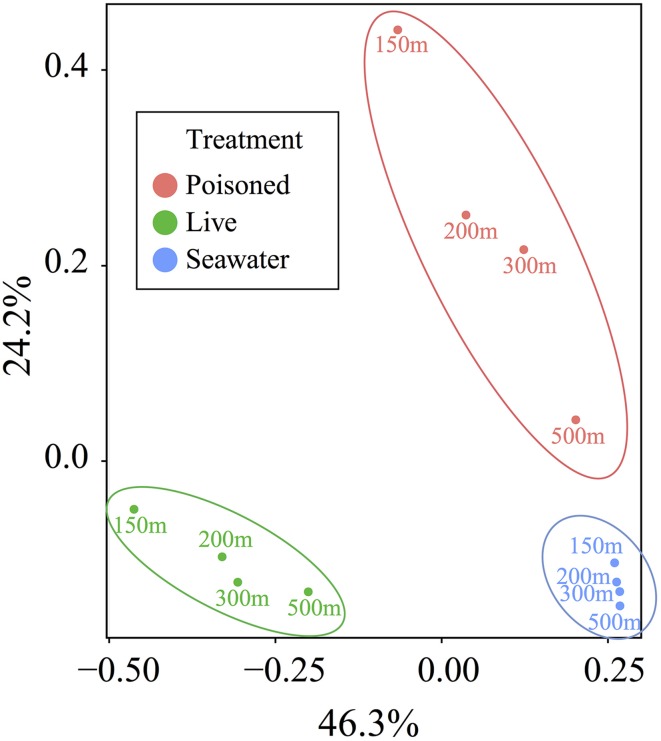
**Principal coordinate analysis of the relative abundance of taxa, as determined by protein-coding genes, in live particle, poisoned particle, and seawater samples**. Samples were grouped into significant clusters according to treatment, as assessed by non-parametric analysis of variance based on dissimilarities (*p* < 0.001).

Principal coordinate analysis of protein-coding genes indicated the presence of distinctive communities in live traps, poisoned traps, and surrounding seawater (*p* < 0.001) and a much lesser effect of depth on community composition (Figure 3). We therefore focused subsequent analyses on the taxonomic and functional differences between live and poisoned sediment traps with minor attention given to depth-related differences.

### Live vs. poisoned sediment trap bacterial assemblages

**Figure 4:**
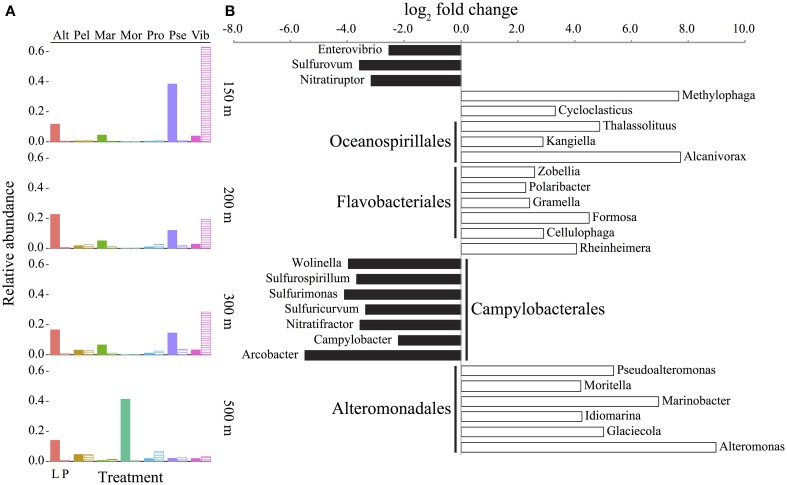
**Abundance and enrichment of bacteria in sediment traps assessed by the taxonomic identification of protein-coding genes. (A)** Genera with at least 2% mean abundance in traps relative to all sequences (including Archaea and Eukarya) in live (L, filled columns) and poisoned (P, striped columns) sediment traps at the indicated depths. Alt, *Alteromonas*; Pel, Candidatus *Pelagibacter*; Mar, *Marinobacter*; Mor, *Moritella*; Pro, *Prochlorococcus*; Pse, *Pseudoalteromonas*; Vib, *Vibrio*. **(B)** Representative bacterial genera (for full figure and dataset see Figure A3 and Dataset A1 in Supplementary Material) that are significantly enriched (FDR < 1%) in live (positive, white) or poisoned (negative, black) sediment traps. Depths were treated as biological replicates to identify statistically significant differences between live and poisoned traps (see Materials and Methods). Order-level identifications are listed in bold for those genera with at least three taxa represented in the comparison.

The most abundant bacterial genera in the live traps were affiliated with the order *Alteromonadales* (*Alteromonas, Marinobacter, Moritella*, and *Pseudoalteromonas*), in contrast to poisoned sediment traps where *Vibrio* was the most highly represented genus (Figure 4A). These trends were consistent across all sampled depths except 500 m, where in the poisoned traps there was a lower more even distribution of different bacterial groups, that included *Pelagibacter* which is typically more abundant in seawater and much less so on particles. The detection of *Prochlorococcus* DNA at 500 m in the poisoned traps may reflect their entrainment on particles or in fecal pellets and subsequent transport into deeper waters, perhaps reflecting the positive correlation between picophytoplankton productivity and their export to the deep sea (Richardson and Jackson, 2007).

**Figure 5:**
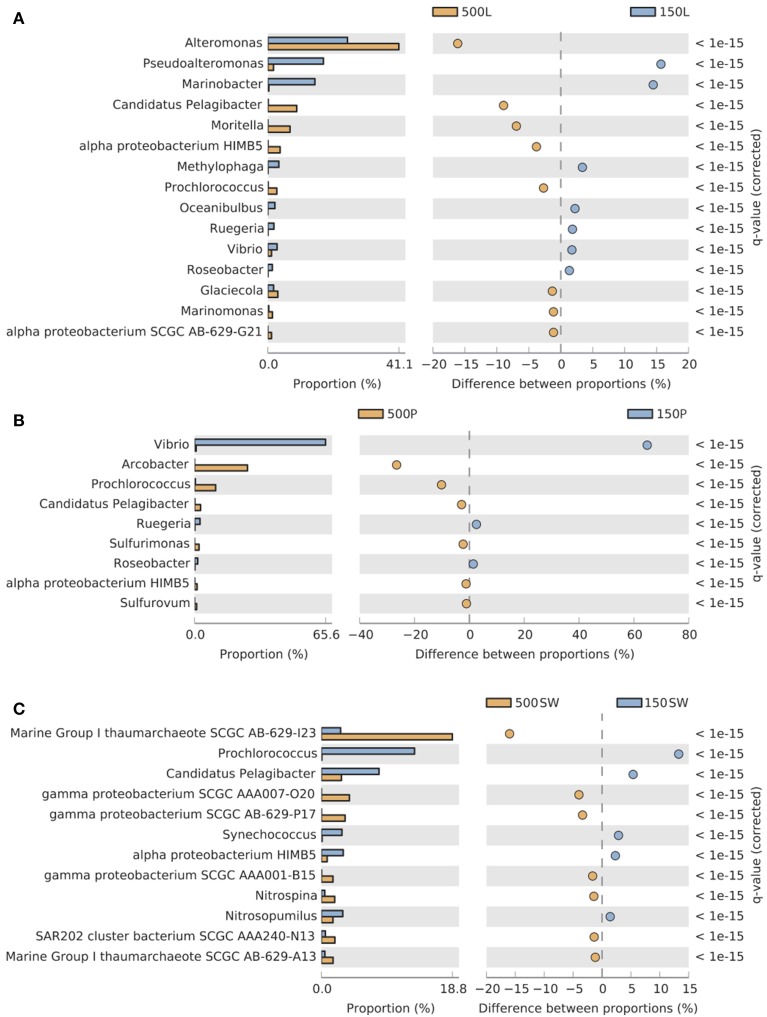
**Pairwise comparisons of selected Bacterial and Archaeal taxa in 150 m vs. 500 m (A) live sediment traps, (B) poisoned sediment traps, and (C) seawater**. Only taxa significantly enriched (FDR < 0.05) at shallow (blue, 150 m) and deep (orange, 500 m) depths with a difference between proportions >1 are shown. The bar plot displays the mean proportion of sequences assigned to each taxon in each sample.

Significant depth-related partitioning in bacterial abundance in the live traps was detected for *Alteromonadales*, with *Alteromonas, Moritella*, and *Glaciecola* significantly enriched at 500 m and *Pseudoalteromonas* and *Marinobacter* enriched at 150 m (Figure 5A). In the poisoned traps, *Vibrio* was also enriched at 150 m (Figure 5B). Depth-related partitioning, also observed in seawater (Figure 5C), may be linked to different lifestyles of the bacteria. *Marinobacter* and *Pseudoalteromonas* are known to associate with eukaryotes (Thomas et al., 2008; Gärdes et al., 2010) and their enrichment at 150 m could be due to their attachment to eukaryotic biomass originating from the euphotic zone. Many alteromonads are typical *r*-strategists, capable of multiplying rapidly in response to nutrient-rich particles and *Alteromonas* species have been found in suspended particle fractions in a variety of ocean basins (Garcia-Martinez et al., 2002; López-Pérez et al., 2012). This may explain their overall high abundance in live traps and enrichment on 500 m particles (Figures 4A, 5A) where they outcompete *k*-strategists like *Pelagibacter* which are generally more abundant in seawater at this depth at Station ALOHA (DeLong et al., 2006).

At all depths, the live traps were significantly enriched in bacterial taxa previously implicated with particle-association, hydrocarbon and dissolved organic matter (DOM) degradation, and eukaryote associations. There was a significant enrichment of *Oceanospirillales, Flavobacteriales*, and *Alteromonadales* in live traps (Figure 4B and Figure A3 in Supplementary Material). Taxa within the *Bacteroidetes*, including *Cytophag*a and *Flavobacteria*, are often found associated with marine snow and phytoplankton blooms in marine environments (DeLong et al., 1993; Teeling et al., 2012). Many *Flavobacteriales* such as *Zobellia, Gramella, Formosa*, and *Cellulophaga* specialize in algal-derived organic matter degradation (Bauer et al., 2006; Bowman, 2006; Mann et al., 2013), which may be an ancestral trait of this group (Thomas et al., 2012). *Methylophaga, Alteromonas, Idiomarina, Glaciecola, Rheinheimera, Polaribacter*, and *Formosa* were also enriched in the live traps, and have also been detected in DOM enrichment events such as phytoplankton blooms and experimental microcosms (Brettar et al., 2006; McCarren et al., 2010; Teeling et al., 2012; von Scheibner et al., 2013). These bacterial types were either growing directly on POM, or on DOM generated *in situ* from POM degradation. *Cycloclasticus, Thalassolituus, Alcanivorax*, and *Marinobacter* were also enriched in live traps (Figure 4B). Species within these genera are often found in high abundance oil-contaminated marine environments, and include some obligately hydrocarbonoclastic species (Yakimov et al., 2007). Enrichment of hydrocarbons on marine POM has long been postulated due their hydrophobicity which may lead to their adsorption on marine POM (Lee et al., 1978; Evans et al., 1990). Recent studies have reported that obligately hydrocarbonoclastic bacteria may associate specifically with phytoplankton as well (Gutierrez et al., 2012). Since hydrocarbons comprise a measurable fraction of carbon in POM (Wakeham and Volkman, 1991) and lipids are the second largest identifiable POM compound class (Lee et al., 2004), these hydrocarbonoclastic species may be participating in the degradation of adsorbed hydrocarbons or those derived directly from eukaryotic plankton (Yoshimura and Hama, 2012; Wei et al., 2013). Several species within *Marinobacter* and *Pseudoalteromonas* are reportedly eukaryote-associated (Thomas et al., 2008; Gärdes et al., 2010) and might be expected to be well-represented in live and poisoned sediment traps. Their enrichment in live traps (Figure 4B), however, suggests that they may be actively growing and degrading their deceased eukaryotic hosts as has been suggested for a several marine symbionts (Grossart, 1999).

The poisoned traps were significantly enriched in chemoautotrophic bacterial types and those with eukaryote-associated lifestyles (Figure 4B and Figures A3, A4 in Supplementary Material). These included epsilon-proteobacteria, particularly *Campylobacterales*, in the poisoned traps (Figure 4B, Figure A3 in Supplementary Material). Presumptive chemoautotrophic sulfur-oxidizing bacteria including *Sulfurimonas, Sulfurovum*, and *Sulfuricurvum* (Campbell et al., 2006) were also enriched in the poisoned traps (Figure 4B, Figure A3 in Supplementary Material). *Sulfurospirillum*, which contains sulfur- and nitrate-reducing heterotrophic species (Stolz et al., 1999), was also enriched in poisoned traps. The presence of sulfur-oxidizing and -reducing taxa in poisoned traps is consistent with previous reports of these metabolic pathways on suspended particles (Fuchsman et al., 2011; Swan et al., 2011). *Sulfurovum* and other epsilon-proteobacterial species have also been found as ectosymbionts of marine invertebrates in both hydrothermal vent and coastal environments (Goffredi, 2010; Ruehland and Dubilier, 2010) suggesting a diverse habitat range for these bacteria on metazoan surfaces in niches where both reduced sulfur compounds and oxygen are readily available. *Nitratiruptor* and *Nitratifractor* are chemolithotrophic hydrogen-oxidizing denitrifiers (Nakagawa et al., 2005) that were also enriched in the poisoned traps (Figure 4B, Figure A3 in Supplementary Material). Denitrification is not considered to be a significant process in well-oxygenated seawater; however it may occur in anoxic microniches within large particles (Karl et al., 1984) or more likely within the intestinal tracts of decaying zooplankton carcasses that have been shown to provide anoxic niches for marine bacteria (Tang et al., 2011; Bickel and Tang, 2014).

Several particle-associated taxa enriched in the poisoned traps were closely related to well-described eukaryote-associated groups, that included *Enterovibrio, Arcobacter, Wolinella*, and *Campylobacter* (Figure 4B). Their presence in poisoned traps is consistent with the detection of diverse eukaryote-associated microbes in seawater and on marine metazoan surfaces (Gugliandolo et al., 2008; Preheim et al., 2011; Turner et al., 2014). Notably, *Vibrionales* genera were similarly enriched in both live and poisoned traps, as compared to seawater (Figure A4 in Supplementary Material). Many *Vibrionales* associate with diverse eukaryotes and are capable of degrading a wide variety of abundant marine biopolymers (Thompson et al., 2004; Takemura et al., 2014). Taken together, these data indicate that bacteria enriched in poisoned traps were most likely associated with eukaryotic surfaces or digestive tracts which is consistent with phyto- and zooplankton detritus constituting the major fraction of marine POM (Simon et al., 2002).

### Live vs. poisoned sediment trap eukaryotic assemblages

The most abundant eukaryotic phyla across all depths in both live and poisoned sediment traps were *Dinoflagellata, Protalveolata, Retaria, Metazoa*, and unclassified *Stramenopiles*, which are likely representative of the composition of sinking particles at Station ALOHA (Figure A5 in Supplementary Material). Protists in class Dinophyceae were also identified microscopically in the 200-m poisoned trap (Figure 1B). Dinoflagellates, radiolarians, and foraminiferan protists, algae, metazoans, and heterotrophic protists were expected to be captured in live and poisoned sediment traps at similar rates.

Differences between live and poisoned traps were most pronounced for *Metazoa* and *Retaria*. The most abundant metazoa across live and poisoned traps were unclassified *Maxillopoda* and a variety of copepod genera (data not shown). Copepod taxa in live traps included *Corycaeus, Clausocalanus*, and *Oithona* while *Scolecithrix* and the ostracod *Conchoecia* were most abundant in poisoned traps (data not shown).

*Retaria* species were slightly more abundant in the poisoned vs. live traps with the most abundant taxa classified as *Acantharia* (poisoned vs. live mean abundance; 1.8% vs. 1.6%) and *Polycystinea* (2.8% vs. 2.4%). A colonial polycystine protist, Sphaerozoum, appeared significantly enriched in the poisoned trap (Figure A6 in Supplementary Material). The silica skeleton of polycystines may be solubilized rapidly in the upper water column at Station ALOHA, with approximately 40% lost within the mesopelagic (Lamborg et al., 2008). Again, *in situ* microbial degradation in the live traps are likely responsible for the differences in eukaryotic taxon abundance between live and poisoned traps. The detection of *Acantharia*, which have strontium sulfate skeletons, in both live and poisoned traps is notable because they are not typically detected in traps without the addition of strontium to the capture solution to inhibit their dissolution (Michaels et al., 1995). This suggests that acantharians detected in the poisoned trap were intact just prior to capture, and that remnants of their DNA in the live trap endured during the 12-day deployment.

### Functional gene categories associated with live and poisoned sediment trap assemblages

KEGG pathways and genes associated with sediment trap DNA were surveyed to functionally profile live vs. poisoned sediment trap metagenome content. A large variety of pathways were significantly enriched in the live vs. poisoned traps, including those associated with motility, amino acid, carbohydrate, and energy metabolism, signal transduction, and cofactors and vitamin biosynthesis (Figure A7 in Supplementary Material). Notably, only the siderophore biosynthesis pathway was enriched in poisoned vs. live sediment trap microbial assemblages. Similarly, siderophore biosynthesis was significantly enriched in poisoned sediment traps compared to seawater (Figure A7 in Supplementary Material). In a recent study, a high frequency of strains containing siderophore biosynthetic genes were linked to eukaryote-associated lifestyles (Cordero et al., 2012), which were enriched in our poisoned sediment traps (Figure A4 in Supplementary Material). These siderophore biosynthesis pathways are likely affiliated with eukaryote-associated taxa since iron acquisition is important for microbial colonization of eukaryotes (Miethke and Marahiel, 2007). Pathways for membrane transport and cell motility were significantly enriched in both live and poisoned sediment traps, compared to seawater (Figure A7 in Supplementary Material).

### Bacterial genes and pathways enriched in the live sediment traps

**Figure 6:**
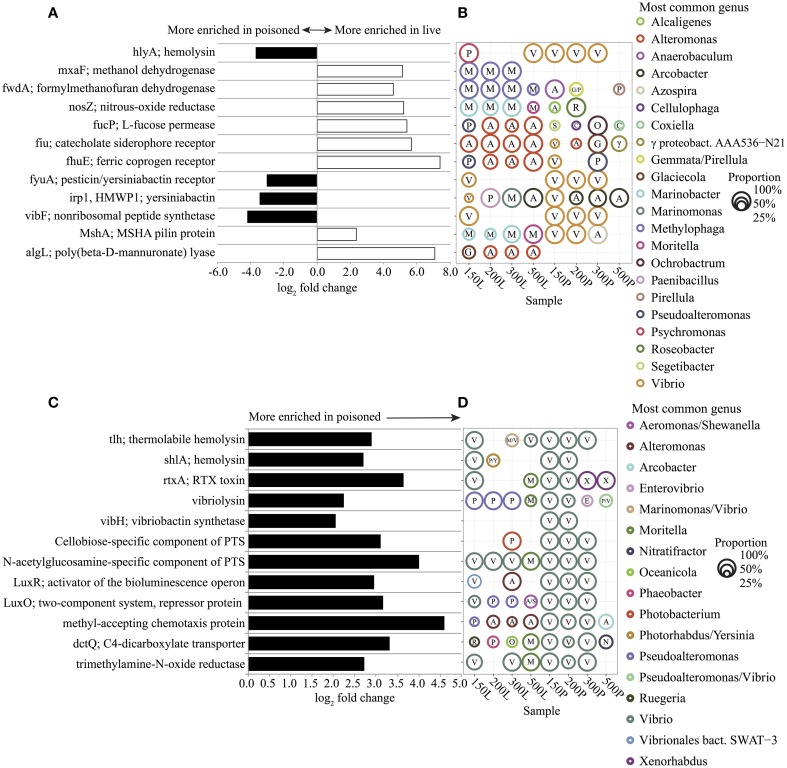
**Representative significantly enriched KEGG genes (FDR < 1%) in live (positive, white) and poisoned (negative, black) sediment traps for comparisons between (A) live and poisoned and (C) poisoned and seawater**. For full gene list see Dataset A2 in Supplementary Material. The most common genus of sequences matching the selected genes and its proportional representation within live (L) and poisoned (P) sediment traps at 150, 200, 300 and 500 meter depths **(B,D)**. The first letter of each genus name is listed within each circle. The circle area represents the proportional representation of each genus within the specified sample. In cases where two genera each represented 50% of the sequences, both are listed.

To explore the potential functions and metabolism associated with *in situ* particle degradation, we surveyed genes and metabolic pathways that were enriched in sediment trap bacterial DNA. Live sediment traps were found to be significantly enriched in genes for TonB-dependent iron transporters (TBDTs), assimilatory and dissimilatory single-carbon compound utilization, and polysaccharide utilization (Figures 6A,B). The majority of sequences matching TBDTs (*fiu, fhuE*) were associated with *Alteromonas spp*. (Figures 6A,B). *Alteromonas-like gene s*equences matching algae- (alginate) and diatom-derived polysaccharide (fucose) utilization were also prevalent (Figures 6A,B). Alginate and fucose utilization genes have been linked to phytoplankton decomposition and assimilation of diatom exopolysaccharides in previous studies (Teeling et al., 2012; Smith et al., 2013). *Methylophaga-like* gene sequences matching key genes of the ribulose monophosphate (RuMP) assimilatory pathway for formaldehyde fixation and detoxification (*hxlA*/*hxlB*) and the tetrahydromethanopterin (THMPT)-dependent dissimilatory formaldehyde oxidation pathway (*FwdABC, ftr, mch*) were also prevalent (Figures 6A,B, Dataset A2 in Supplementary Material). These data are consistent with previous microcosm experiments that showed the enrichment of *Alteromonadaceae* and *Methylophaga* phylotypes, as well as their expressed genes, including *Alteromonadaceae* TBDTs and *Methylophaga*-like key enzymes of the RuMP pathway and the THMPT-dependent pathway (Pinhassi et al., 2004; Neufeld et al., 2008; McCarren et al., 2010). Potential growth of these groups on DOM generated in the live trap by POM breakdown is also consistent with previous zooplankton and phytoplankton degradation studies that have demonstrated rapid accumulation of DOM over the course of just a few days during POM diagenesis (Yoshimura et al., 2009; Yoshimura and Hama, 2012).

Other bacterial genes enriched in the live sediment traps included those associated with denitrification from nitrate to N_2_ (nitrate reductase; *narG*, nitrate reductase; *nirS*, nitric oxide reductase; *norB*, and nitrous oxide reductase; *nosZ*). (Figures 6A,B, Dataset A2 in Supplementary Material) Genes encoding a mannose-sensitive hemagglutinin (MSHA) pilus were found associated with *Marinobacter* at depths between 150–300 m, and *Moritella* at 500 m (Figures 6A,B). The MSHA pilus has been implicated in attachment to animal surfaces in *Vibrio* and *Pseudoalteromonas* (Chiavelli et al., 2001; Dalisay, 2006) and it may also play a role in attachment by *Moritella* and *Marinobacter*, which have previously been shown to associate with eukaryotes (Gärdes et al., 2011; Tunsjø et al., 2011). The *pilA* gene encoding the pilin protein of a novel chitin-regulated pilus (ChRP;K02650) along with a chitin-binding protein gene (CBP; K03933) were also highly enriched in live traps (Dataset A2 in Supplementary Material). In addition, a variety of heavy-metal resistance genes associated with *Alteromonadales* were enriched in the live traps (Figures A8A,B and Dataset A2 in Supplementary Material). The *czcABCD* genes encode a heavy metal efflux pump involved in resistance to cobalt, zinc, and cadmium that were affiliated with *Alteromonas*. *CusAB* and *cusRS* encode copper efflux proteins and copper two-component sensor systems, respectively, that were affiliated with *Alteromonas, Glaciecola*, and *Marinobacter*. The genes involved in mercury resistance (*merABR*) and transport (*merTP*) were affiliated with *Alteromonas* and *Marinobacter*. The enrichment of metal resistance genes in the poisoned traps may be linked to their growth on particles, which are known to concentrate heavy metals (Puig et al., 1999).

**Figure 7:**
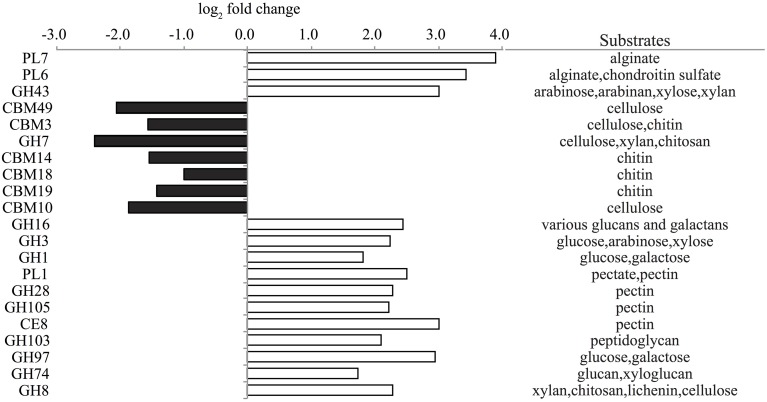
**Representative significantly enriched CAZy families (FDR < 1%) in live (positive, white) and poisoned (negative, black) sediment traps along with their potential substrates**. For full family list, see Dataset A4 in Supplementary Material. PL, polysaccharide lyase; GH, glycoside hydrolase; CBM, carbohydrate-binding modules; CE, carbohydrate esterase.

To further evaluate the potential for carbohydrate degradation capabilities of trap associated microbial assemblages, peptide encoding sequences were compared to a carbohydrate-active enzymes database (CAZymes) (Yin et al., 2012). A large number of glycoside hydrolase (GH) families associated with polysaccharide degradation found in algae and bacterial cell walls including arabinose, pectin, cellulose, and peptidoglycan were significantly enriched in the live traps (Figure 7). Polysaccharide lysase (PL) families 6,7 and 1 and carbohydrate esterase family 8 complement the degradation potential of GHs (Cantarel et al., 2009) and potentially may enhance the degradation of the algal substrates alginate and pectin in live traps (Figure 7). Several carbohydrate-binding module (CBM) families targeting chitin (e.g., CBM1, CBM14, and CBM18) were also enriched in live traps (Dataset A4 in Supplementary Material).

### Bacterial genes and pathways enriched in the poisoned sediment traps

Poisoned sediment trap particles were significantly enriched in a variety of iron-scavenging genes and virulence factors, primarily associated with *Vibrio* spp. (Figures 6A–D). *Vibrio* spp. are known to engage in pathogenic, symbiotic, and saprophytic associations with a wide variety of eukaryotes in the marine environment (Takemura et al., 2014). *Vibrio*-like *genes* for carbohydrate uptake and chemotaxis, supporting eukaryote-associated lifestyles, were significantly enriched in poisoned sediment traps (Figures 6C,D). *Vibrio* genes for chitin utilization, including those associated with sensing, attachment, degradation, and uptake of chitin derivatives (Keyhani and Roseman, 1999; Beier and Bertilsson, 2013) were also enriched in the poisoned sediment traps. These included a methyl-accepting chemotaxis protein that mediates a chemotactic response to N-acetylglucosamine (GlcNAc) (Meibom et al., 2004) and a CBP (K03933; Cazy AA10) that mediates *Vibrio* spp. attachment to chitin surfaces and enzymatically cleaves chitin, which were both highly enriched in poisoned traps (Figures 6C,D and Dataset A2 in Supplementary Material) (Vaaje-Kolstad et al., 2010; Frederiksen et al., 2013). Transporters mediating uptake of cellobiose and GlcNAc were also highly enriched in poisoned traps (Figures 6C,D). Together, these data support the association of Vibrio in poisoned traps with chitin substrates, and are consistent with the presence of copepods detected in DNA analyses and in optical microscopy (Figure 1H) in the same traps.

A variety of genes involved in quorum sensing and anaerobic metabolism, also associated with *Vibrio* spp., were significantly enriched in poisoned traps (Figures 6C,D). They included the *luxS*-*luxP*/*Q* quorum-sensing system, the *luxOR* bioluminescence regulators, trimethylamine *N*-oxide (TMAO) reductase, and the TMAO two-component regulatory sensors (*torR/S*) (Figures 6C,D and Dataset A2 in Supplementary Material). TMAO is an abundant osmolyte found in the tissues of marine eukaryotes that can be utilized aerobically and anaerobically by diverse marine bacteria (Barrett and Kwan, 1985; Proctor and Gunsalus, 2000; Sun et al., 2011). Enrichment of TMAO genes is thus consistent with eukaryotic association in live animals or sedimenting particles that entered the poisoned traps.

Comparisons with the CAZyme database revealed that a variety of CBM families targeting cellulose and chitin were enriched in the poisoned traps (Figure 7). CBMs complement the activity of other enzymes by promoting extended interactions with substrates (Cantarel et al., 2009). Further, GH families 7 and 19 catalyze the degradation of cellulose and chitin and were highly enriched in poisoned traps as compared to live traps and seawater, respectively (Figure 7 and Dataset A4 in Supplementary Material). Auxiliary activity (AA) family 10, formerly classified as CBM family 33, capable of cleaving chitin and cellulose (Aachmann et al., 2012) was enriched in both live and poisoned traps, as compared to seawater (Dataset A4 in Supplementary Material). These data support KEGG functional profiles indicating the potential for chitin degradation in both live and poisoned sediment traps.

## Conclusion

Sinking particles represent the primary vehicles of organic carbon flux from surface waters to the deep ocean (Volk and Hoffert, 1985), yet to date, few data are available on the specific microbes and metabolic pathways responsible for POM degradation throughout the water column. There is a general consensus that particles represent hotspots of microbial activity in the ocean (Karl and Knauer, 1984; Turley and Mackie, 1994; Crump et al., 1999; Bochdansky et al., 2010; Smith et al., 2013), but the nature of those processes and microorganisms responsible still need to be better described.

In this study, sediment trap metagenomic analyses revealed dramatic differences in the taxonomic diversity and functional potential of microbes associated with sinking particles in poisoned sediment traps, compared to those that grew *in situ* in live traps. Both live and poisoned sediment trap microbial assemblages were distinctly different from those found in seawater, which is consistent with the conclusions of several recent studies (LeCleir et al., 2013; Smith et al., 2013). Live particle-trap assemblages shared many similarities with communities found in microcosm DOM enrichments, with the added dimension of known particle-associated bacteria (e.g., *Flavobacteriales*) and potentially eukaryote-associated bacteria (e.g., *Pseudoalteromonas* and *Marinobacter*). The functional gene content in live traps pointed to the potential for growth by alteromonads on labile DOM produced *in situ* from sinking POM. Apparently, the contained environment within the sediment trap acted similarly to microcosm enrichment experiments, where fast-growing copiotrophic bacteria out-competed the particle- and eukaryote-associated bacteria for the nutrients available in the trap. The poisoned sediment trap-associated metagenomic analyses provided a clear contrast to live traps and presumably reflected the biological material and microbial assemblages associated with sinking particles. The differences in composition between live and poisoned traps were much greater than depth associated differences, consistent with previous studies of suspended particulates found in oxygen minimum zones (Ganesh et al., 2014).

In total, these findings are consistent with a previous study that suggested initial particle-colonizers are surface-colonizing (or eukaryote-associated) specialists (LeCleir et al., 2013). Our metagenomic data further indicated that microbes in poisoned sediment traps were often associated with eukaryotic surfaces and intestinal tracts as symbionts, pathogens, or saprophytes. Some of these eukaryote-associated bacteria may alternate between symbiotic to pathogenic or saprophytic lifestyles, as has been shown for some phytoplankton symbionts (Grossart, 1999; Seyedsayamdost et al., 2011). The functional gene content in poisoned traps, which included a variety of genes involved in virulence, anaerobic metabolism, attachment to chitinaceous surfaces, and chitin degradation were consistent with this conclusion. Notably, genes for attachment to chitinaceous surfaces and anaerobic metabolism were also detected in live traps, though they were associated with a different set of microbial taxa. Thus, eukaryote-associated communities captured in live and poisoned traps differed, most likely due to bacterial growth in the live trap.

Our data also provide new perspective on the taxonomic identity of the particulate matter itself, namely the eukaryotic taxa that contribute to the complex mixture of detritus and minerals that make up marine particles. While previous studies have reported marine particles as consisting of eukaryote-derived detritus, the analyses we report here suggests that specific interactions between eukaryotes and bacteria may be centrally important in the transport and degradation processes associated with sinking POM. The presence of chitinaceous surfaces provides a habitat for a specialized bacterial community adapted to sense, attach, degrade, and take up chitin derivatives. These same habitats appeared to coincide with the development of copiotrophs known to respond rapidly to labile DOM inputs. The probable sources of labile DOM include turnover of phytoplankton captured in the traps, the excretions of swimming zooplankton, and substrates from the degradation of captured chitinaceous detritus.

This study provides a baseline for understanding microbial community assemblages and metabolic activities associated with the transport and degradation of sinking POM. To date, gene expression associated with POM degradation has not yet been reported, partly due to the technical difficulties associated with preserving RNA *in situ*. Future metatranscriptomic analyses have the potential to identify those metabolic pathways that are expressed *in situ* on sinking particles and help to define the processes that actively drive particle degradation in the ocean's interior. Finer scale studies of particle transport and degradation should also help to define hypothetical successional cascades that reflect sequential processing of POM to DOM during its transport to the deep-sea.

## Author contributions

KF, ED, DK designed research. JE contributed bioinformatics tools. KF performed research. TS performed microscopy. KF, JE, and ED analyzed data. KF, ED, and DK wrote the paper.

### Conflict of interest statement

The authors declare that the research was conducted in the absence of any commercial or financial relationships that could be construed as a potential conflict of interest.
