# Supplementary material for: Microbial community structure and function on sinking particles in the North Pacific Subtropical Gyre
Source: Front Microbiol. 2015 May 19;6:469. doi: 10.3389/fmicb.2015.00469 (PMC4436931; doi:10.3389/fmicb.2015.00469)

Supplementary Appendix:
Microbial community structure and function on sinking particles
in the North Pacific Subtropical Gyre

Kristina M. Fontanez[1], John M. Eppley[1,2,3], Ty J. Samo[2,3], David M. Karl[2,3] and Edward F. DeLong[1,2,3]*

[1]Department of Civil and Environmental Engineering, Massachusetts Institute of Technology, Cambridge, MA 02139; [2]Department of Oceanography, School of Ocean and Earth Science and Technology, University of Hawaii, Honolulu, HI 96822; [3] Daniel K. Inouye Center for Microbial Oceanography, Research and Education, University of Hawaii, 1950 East-West Road, Honolulu, HI 96822

*Correspondence: Edward F. DeLong, Department of Oceanography, School of Ocean and Earth Science and Technology, University of Hawaii, Honolulu, HI 96822. Tel: (808) 956-0563 E-mail: edelong@hawaii.edu

Index of Supplementary Appendix

1. Supplementary Text
2. Supplementary References
3. Legends for Supplementary Datasets
4. Supplementary Tables
Table A1. Sequencing data and database comparisons for sediment traps and seawater samples.
5. Supplementary Figures
Figure A1. The location of sediment traps in the water column in relation to hydrography at Station ALOHA.
Figure A2. Abundance of the three major archaeal phyla.
Figure A3. RefSeq bacterial genera that are significantly enriched (FDR < 1% and > 2 $\log_2$ fold change) in live (positive, white) or poisoned (negative, black) sediment traps.
Figure A4. Bacterial genera that are significantly enriched (FDR < 1% and at least 4 $\log_2$ fold change in abundance) in a comparison among live traps, poisoned traps and surrounding seawater according to the taxonomic identification of protein-coding sequences.
Figure A5. Abundance of eukaryotic OTUs, by phylum, relative to all sequences (including Bacteria and Archaea) in live (L, white) and poisoned (P, black) sediment traps as determined by the small subunit ribosomal RNA genes.
Figure A6. Microbial genera that are significantly enriched (FDR < 1%) in live (positive) or poisoned (negative) sediment traps, according to small subunit ribosomal RNA genes.
Figure A7. Functional pathways that are significantly enriched (FDR < 1%) in a comparison among live traps, poisoned traps and surrounding seawater, as classified by KEGG level 3.
Figure A8. Enrichment of metal resistance genes on live marine particles.

1. Supplementary Text

Methods

Sample collection. Sediment trap samples were collected during the HOE-DYLAN 5 (KM1215) cruise. Sediment traps sampled for metagenomics (150 m, 200 m, 300 m and 500 m) were deployed at Station ALOHA on July 14, 2012 at 4:00 AM (HST) and recovered at 5:30 AM (HST) on July 26, 2012 after drifting 75 nautical miles. Seawater at parallel depths was collected during the HOE-DYLAN 9 (KM1219) cruise using a CTD rosette on lowering S2C28 on August 26, 2012. Approximately 20 L of seawater per depth was filtered through a 5 μm Durapore (EMD Millipore, Billerica, MA) filter and microbial communities collected on a 0.2 μm Sterivex (EMD Millipore) using a peristaltic pump. The sample was preserved with 1.5 mL RNAlater (Ambion, Carlsbad, CA) and stored at -80°C until DNA isolation.

Sediment trap solutions. Live sediment traps were filled with 0.2 μm-filtered surface seawater mixed with sodium chloride (33.8 g/L) (brine solution), magnesium chloride (14.3 g/L) and potassium chloride (0.93 g/L) (Knauer et al., 1984). Poisoned sediment traps were filled with a combination of dilute and full-strength homebrew RNAlater. The recipe for homebrew RNAlater is as follows: First, combine 40 mL 0.5M EDTA, 25 mL 1M sodium citrate, and 700 grams ammonium sulfate with 935 mL ultrapure water. Stir on low to moderate heat until the ammonium sulfate dissolves completely. Allow to cool for about 30 minutes. Next, adjust pH to 5.2 using sulfuric acid. Finally, remove particulates and sterilize using a 0.2 μm filter. The poisoned trap tubes were first filled up to 1 L with a diluted (1.05 g/mL) homebrew RNAlater. Then, 500 mL of full-strength homebrew RNAlater was slowly pumped into the bottom of the trap using a peristaltic pump. Finally, the dilute homebrew RNAlater was used to carefully bring up the total volume to 2 cm below the top of the trap tube, avoiding disturbance of the denser bottom layer. A density interface was visible between the dense full-strength RNAlater at the bottom of the tube and the more dilute RNAlater at the top. Finally, the traps were fitted with a 335 um Nitex screen below the topmost baffle in order to exclude larger zooplankton.

Sediment trap processing. All sediment traps were pre-processed prior to filtration. First, the baffle and 335 μm Nitex screen were removed. Next, the interface between overlying seawater and the high density solution in the trap was visualized and marked. Then, overlying seawater was removed from each trap using a peristaltic pump. The remaining trap slurry was filtered through a 0.2 μm Sterivex filter, preserved with 1.5 mL RNAlater (Ambion), and stored at -80°C until DNA isolation. In this study, the average volume filtered was 1.61 and 1.70 Liters for live and poisoned sediment traps, respectively.

DNA isolation. The 1.5 mL of RNAlater was expunged from stored Sterivex filters using a luer syringe. Sterivex filter cartridges were opened using ethanol-flamed razor blades, gripped with locking pliers. Hot razor blades were firmly pressed around the circumference of the sterivex on the male luer side until a clean slice through the plastic was achieved. Multiple razor blades were flamed and applied in quick succession to completely slice through the plastic. Then, the plastic casing around the filter was removed by gripping the female luer port and pulling gently so as not to disturb the filter. Finally, an ethanol-flamed razor blade was used to slice around the

circumference of the filter, at both ends, and bisect it resulting in 2 equally sized rectangular filter pieces. Filter pieces were then placed in sterile falcon tubes and the unused half filter was stored in a cryovial at -80°C. The following describes the modifications to the Mobio Powerwater DNA Isolation kit (Mobio, Carlsbad, CA) protocol used to extract whole community DNA from the half filters. PW1 solution was warmed to 55°C for 10 minutes. The half filter was added to the Powerwater bead tube with 1.2 mL of warmed PW1 and vortexed for a total of 10 minutes. Tubes were centrifuged at 4000xg for 1 minute at room temperature. The supernatant was transferred to a 1.7 mL tube, placing the pipet tip down into the beads to draw up all of the supernatant. The supernatant was centrifuged at 13,000xg for 1 minute. Avoiding the pellet, the lysate was split among two new tubes with no more than 650 μL per tube. Next, 200 μL PW2 was added to each tube. The standard protocol was followed for the rest of the isolation, except that DNA was eluted in 25 μL PW6 using a low-volume elution filter (Mobio) and quantified using the PicoGreen dsDNA assay (Invitrogen, Carlsbad, CA).

Library preparation and sequencing. Library preparation followed the Nextera XT DNA sample preparation protocol (Illumina, San Diego, CA, USA). Samples were dual-indexed according to the low-plexity pooling guidelines (Nextera XT Sample Preparation Guide 15031941_C) and 10 samples pooled per sequencing run (2x300 bp) on a MiSeq instrument using MiSeq reagent kit v3 (Illumina). Sequencing and quality control followed the manufacturer's recommendations.

Sequence analysis and annotation. Sequencing and annotation statistics for sediment trap and seawater samples are summarized in **Table A1**. Illumina sequences were filtered with Trimmomatic v. 0.27 setting ILLUMINACLIP::2:40:15 (Lohse et al., 2012), to remove primers and adapters. Next, PandaSeq v. 2.4 (Masella et al., 2012) was used to join paired reads. Unjoined read pairs were joined with 6 N's and tracked along with paired reads. All reads were trimmed to remove low quality bases (< 5) from the ends. Reads below the PandSeq score cutoff of 0.32, containing fewer than 40 bases, or in which > 90% of bases were a single nucleotide, were removed. Remaining reads were filtered using SortMeRNA v. 1.7 (galaxy tool revision 2e7f0da431e3) to identify rRNA-containing reads (Kopylova et al., 2012) using the following databases constructed from the Silva SSU_LSU (version 111) and the Rfam 5/5.8s (version 11.0): rfam-5.8s, rfam-5s, silva-archaea-16s, silva-archaea-23s, silva-bacteria-16s, silva-bacteria-23, silva-eukarya-18s, and silva-eukarya-28s. Taxonomy of rRNA and non-rRNA reads was determined by comparison against SILVA release 115 and the NCBI RefSeq release 61 databases, respectively, using lastal v. 418 (Kiełbasa et al., 2011). Function of non-rRNA reads was determined by comparison with the September 2013 version of the Kyoto Encyclopedia of Genes and Genomes (KEGG) (Kanehisa and Goto, 2000) database using lastal (Kiełbasa et al., 2011) and the March 22, 2013 version of the Carbohydrate Active Enzyme (CAZy) database (Cantarel et al., 2009) using HMMER3.0 (Eddy, 2011) and hidden Markov models (HMM) of CAZy signature domains (Bolger et al., 2014; Yin et al., 2012). To identify significant matches lastal minimum alignment scores (-e) of 40 and 100 were used for rRNA reads and non-rRNA reads, respectively. Additional protein search parameters included -b 1 -x 15 -y 7 -z 25 -F 15 -u 2 -Q 0. For sequences that matched equally well according to lastal score to multiple reference genes, sequences were assigned to the reference gene that was most frequently identified in the dataset. HMM searches of the CAZy signature domains utilized a custom script that incorporated translation of non-rRNA reads to amino acids using EMBOSS (Rice et al., 2000), comparison

against HMMs using HMMER3 (Eddy, 2011), and two parallel filters. The alpha filter kept hits > 80 amino acids and e-value < 1e-5. The beta filter kept hits that cover at least 30% of the HMM with an e-value < 1e-3. The resulting hits were merged and the best scoring hit for each read was retained. For sequences with multiple best hits, the sequences were assigned to the HMM that was most frequently identified in the dataset.

Statistical analyses. All metagenomic sequences were normalized and variance stabilized using the regularized log transformation in DESeq2 setting blind=FALSE to incorporate the experimental design in which all four depths belonging to a treatment are modeled as biological replicates (Anders and Huber, 2010). This function transforms the data to the log2 scale using the Tikhonov/ridge regularization in order to address the issues of varying sequencing library size and feature counts that vary more than expected under a Poisson model (overdispersion). Transformed data were converted to expected counts using 2^(transformed value) and used to create relative abundance plots of taxa, pathways and genes using the phyloseq R package (McMurdie and Holmes, 2013). Ordination of normalized sequences used principal coordinate analysis with bray-curtis distance in phyloseq (McMurdie and Holmes, 2013). Significance ($p < 0.05$) of clusters was determined using non-parametric analysis of variance based on dissimilarities (Adonis function) in the vegan R package (Dixon, 2003). A negative binomial Wald test in DESeq2 was used to identify statistically significant differences in taxonomic and functional non-normalized gene counts among live traps, poisoned traps, and seawater. As replicates of sediment traps at each depth were not available, all four depths belonging to a treatment were modeled as biological replicates. A false discovery rate threshold of 0.01 (Benjamini and Hochberg, 1995) was used for detecting differentially abundant taxa or functions. For statistical validation of depth-specific taxonomic differences, Fisher's exact test as implemented in the STAMP v2.01 program (Parks and Beiko, 2010) was used for pairwise comparisons of 150 meter versus 500 meter RefSeq-identified non-normalized taxa within treatment types. A false discovery rate threshold of 0.05 (Benjamini and Hochberg, 1995) and a difference between proportions cutoff of 1 were used to assess statistical and biological significance, respectively.

2. Supplementary References

Anders, S., and Huber, W. (2010). Differential expression analysis for sequence count data. *Genome Biol.* 11, R106. doi:10.1186/gb-2010-11-10-r106.

Benjamini, Y., and Hochberg, Y. (1995). Controlling the false discovery rate: a practical and powerful approach to multiple testing. *J. Roy. Stat. Soc. Series B Stat. Methodol.*, 289–300.

Bolger, A. M., Lohse, M., and Usadel, B. (2014). Trimmomatic: a flexible trimmer for Illumina sequence data. *Bioinformatics* 30, 2114–2120. doi:10.1093/bioinformatics/btu170.

Cantarel, B. L., Coutinho, P. M., Rancurel, C., Bernard, T., Lombard, V., and Henrissat, B. (2009). The Carbohydrate-Active EnZymes database (CAZy): an expert resource for Glycogenomics. *Nucleic Acids Res.* 37, D233–8. doi:10.1093/nar/gkn663.

Dixon, P. (2003). VEGAN, a package of R functions for community ecology. *J. Veg. Sci.* 14, 927–930.

Eddy, S. R. (2011). Accelerated profile HMM searches. *PLoS Comput. Biol.* 7, e1002195.

Kanehisa, M., and Goto, S. (2000). KEGG: Kyoto encyclopedia of genes and genomes. *Nucleic Acids Res.* 28, 27–30.

Kiełbasa, S. M., Wan, R., Sato, K., Horton, P., and Frith, M. C. (2011). Adaptive seeds tame genomic sequence comparison. *Genome Res.* 21, 487–493. doi:10.1101/gr.113985.110.

Knauer, G. A., Karl, D. M., Martin, J. H., and Hunter, C. N. (1984). In situ effects of selected preservatives on total carbon, nitrogen and metals collected in sediment traps. *J. Mar. Res.* 42, 445–462. doi:10.1357/002224084788502710.

Kopylova, E., Noé, L., and Touzet, H. (2012). SortMeRNA: fast and accurate filtering of ribosomal RNAs in metatranscriptomic data. *Bioinformatics* 28, 3211–3217. doi:10.1093/bioinformatics/bts611.

Lohse, M., Bolger, A. M., Nagel, A., Fernie, A. R., Lunn, J. E., Stitt, M., and Usadel, B. (2012). RobiNA: a user-friendly, integrated software solution for RNA-Seq-based transcriptomics. *Nucleic Acids Res.* 40, W622–7. doi:10.1093/nar/gks540.

Masella, A. P., Bartram, A. K., Truszkowski, J. M., Brown, D. G., and Neufeld, J. D. (2012). PANDAseq: PAired-eND Assembler for Illumina sequences. *BMC Bioinformatics* 13, 31. doi:10.1186/1471-2105-13-31.

McMurdie, P. J., and Holmes, S. (2013). phyloseq: an R package for reproducible interactive analysis and graphics of microbiome census data. *PLoS ONE* 8, e61217. doi:10.1371/journal.pone.0061217.

Parks, D. H., and Beiko, R. G. (2010). Identifying biologically relevant differences between

metagenomic communities. *Bioinformatics* 26, 715–721. doi:10.1093/bioinformatics/btq041.

Rice, P., Longden, I., and Bleasby, A. (2000). EMBOSS: The European Molecular Biology Open Software Suite. *Trends Genet.* 16, 276–277. doi:10.1016/S0168-9525(00)02024-2.

Yin, Y., Mao, X., Yang, J., Chen, X., Mao, F., and Xu, Y. (2012). dbCAN: a web resource for automated carbohydrate-active enzyme annotation. *Nucleic Acids Res.* 40, W445–W451. doi:10.1093/nar/gks479.

3. Legends for Supplementary Datasets

Dataset A1. Results of significance tests for RefSeq-classified OTUs.
Dataset A2. Results of significance tests for KEGG-classified genes.
Dataset A3. Results of significance tests for Silva-classified SSU OTUs.
Dataset A4. Results of significance tests for Cazy-classified gene families.

4. Supplementary Tables

Table A1. Sequencing data and database comparisons for sediment traps and seawater samples. Yellow highlighted rows indicate the stark differences between the percentage of sequences identifiable in the poisoned traps (P) versus those identifiable in seawater (SW) and in the live traps (L) for ribosomal RNA (Silva) and protein (KEGG,CAZy) databases. Hawaii Ocean Experiment - Dynamics of Light and Nutrients (HOE-DYLAN). HD5=HOE-DYLAN V, HD9=HOE-DYLAN IX.0.2=0.2 micron-filtered seawater

Cruise and Sample ID	Filtered reads	Mean length (bp)	rRNA reads	non-rRNA reads	% rRNA	unique rRNA reads matching Silva	% of rRNA reads with significant Silva hits	unique non-rRNA reads matching RefSeq	% of non-rRNA reads with signficant RefSeq hits	unique non-rRNA reads matching KEGG	% of non-rRNA reads with significant KEGG hits	unique non-rRNA reads matching CAZy	% of non-rRNA reads with significant CAZy hits
HD5 150L	2,480,180	281	14,532	2,465,648	0.59%	2,216	15%	1,875,591	76%	1,708,939	69%	50,012	2.0%
HD5 200L	564,756	412	3,428	561,328	0.61%	672	20%	443,199	78%	412,989	74%	13,796	2.5%
HD5 300L	1,584,828	263	9,147	1,575,681	0.58%	1,177	13%	1,094,925	69%	974,174	62%	26,918	1.7%
HD5 500L	1,531,861	231	8,703	1,523,158	0.57%	1,096	13%	570,294	37%	505,074	33%	17,160	1.1%
HD5 150P	3,500,721	204	39,077	3,461,644	1.12%	2,827	7%	714,348	21%	580,399	17%	20,977	0.6%
HD5 200P	2,024,333	211	10,678	2,013,655	0.53%	1,035	10%	224,115	11%	191,406	10%	8,855	0.4%
HD5 300P	459,246	213	3,863	455,383	0.84%	344	9%	55,569	12%	47,020	10%	2,032	0.4%
HD5 500P	1,253,503	253	9,067	1,244,436	0.72%	1,230	14%	181,440	15%	164,270	13%	6,727	0.5%
HD9 SW 150-0.2	1,268,822	423	4,244	1,264,578	0.33%	888	21%	541,818	43%	468,810	37%	24,083	1.9%
HD9 SW 200-0.2	4,367,790	340	15,670	4,352,120	0.36%	3,910	25%	2,279,283	52%	1,978,450	45%	94,864	2.2%
HD9 SW 300-0.2	1,212,964	363	3,514	1,209,450	0.3%	750	21%	603,471	50%	514,606	43%	24,936	2.1%
HD9 SW 500-0.2	905,712	375	2,727	902,985	0.3%	591	22%	552,076	61%	466,189	52%	20,456	2.3%

5. Supplementary Figures

Figure A1. The location of sediment traps in the water column in relation to hydrography at Station ALOHA. The traps used for metagenomics were sampled at 150 m, 200 m, 300 m and 500 m depths and deployed for 12 days from 7/14/12 to 7/26/12.

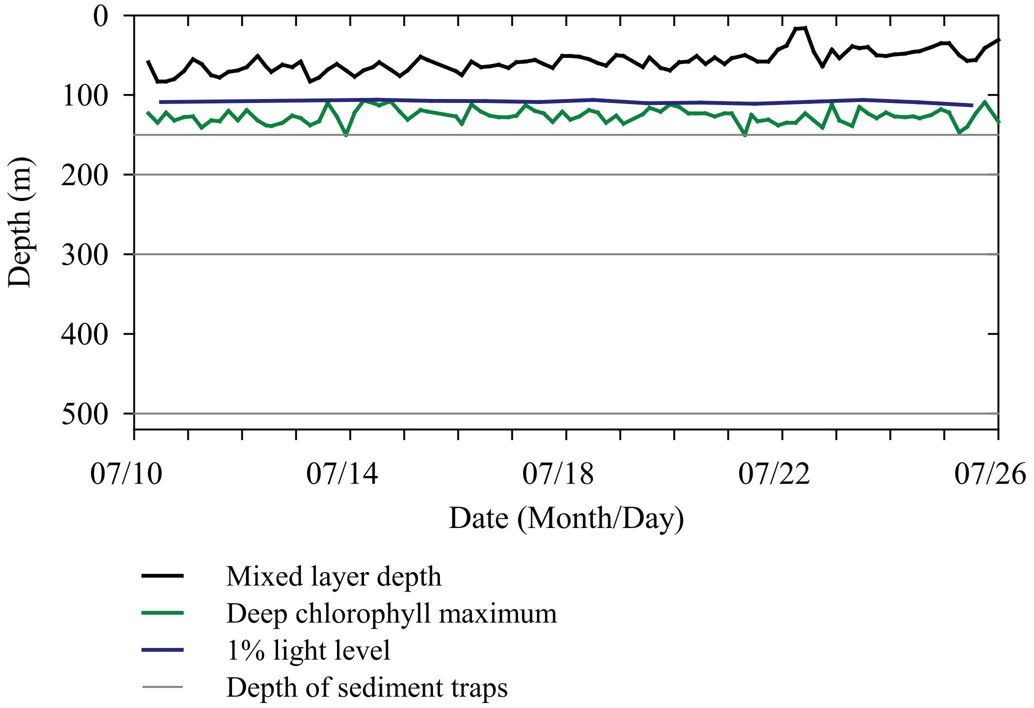

Figure A2. Abundance of the three major archaeal phyla. Abundances are relative to all sequences (Archaea, Bacteria and Eukarya) in live and poisoned sediment traps, as well as surrounding seawater, at the indicated depths according to the taxonomic identifications of protein-coding sequences.

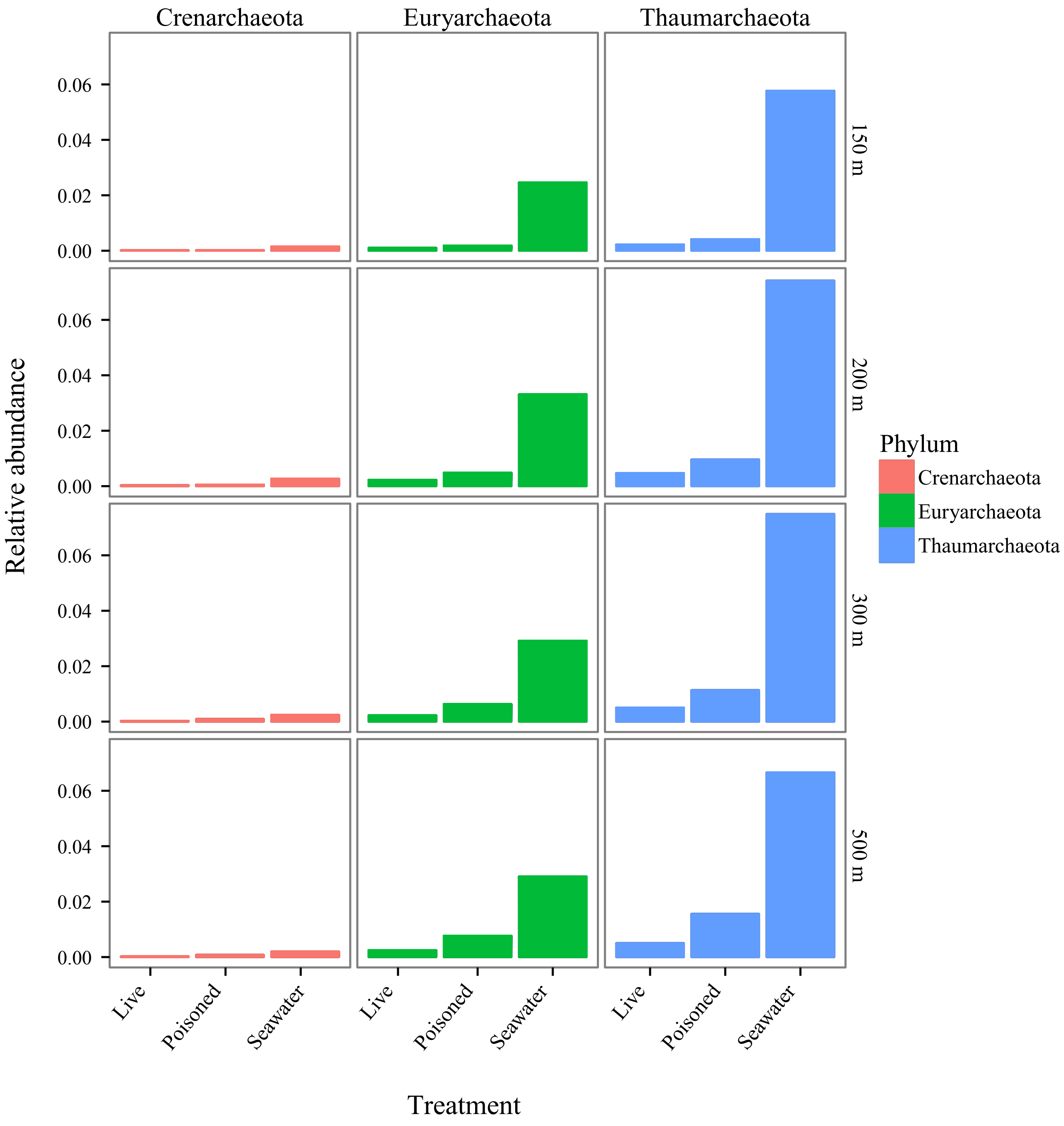

Figure A3. RefSeq bacterial genera that are significantly enriched (FDR < 1% and >2 log_2 fold change) in live (positive, white) or poisoned (negative, black) sediment traps. For full gene list see Dataset A1 in Appendix. Order-level identifications are listed for those genera with at least 5 taxa represented in the comparison.

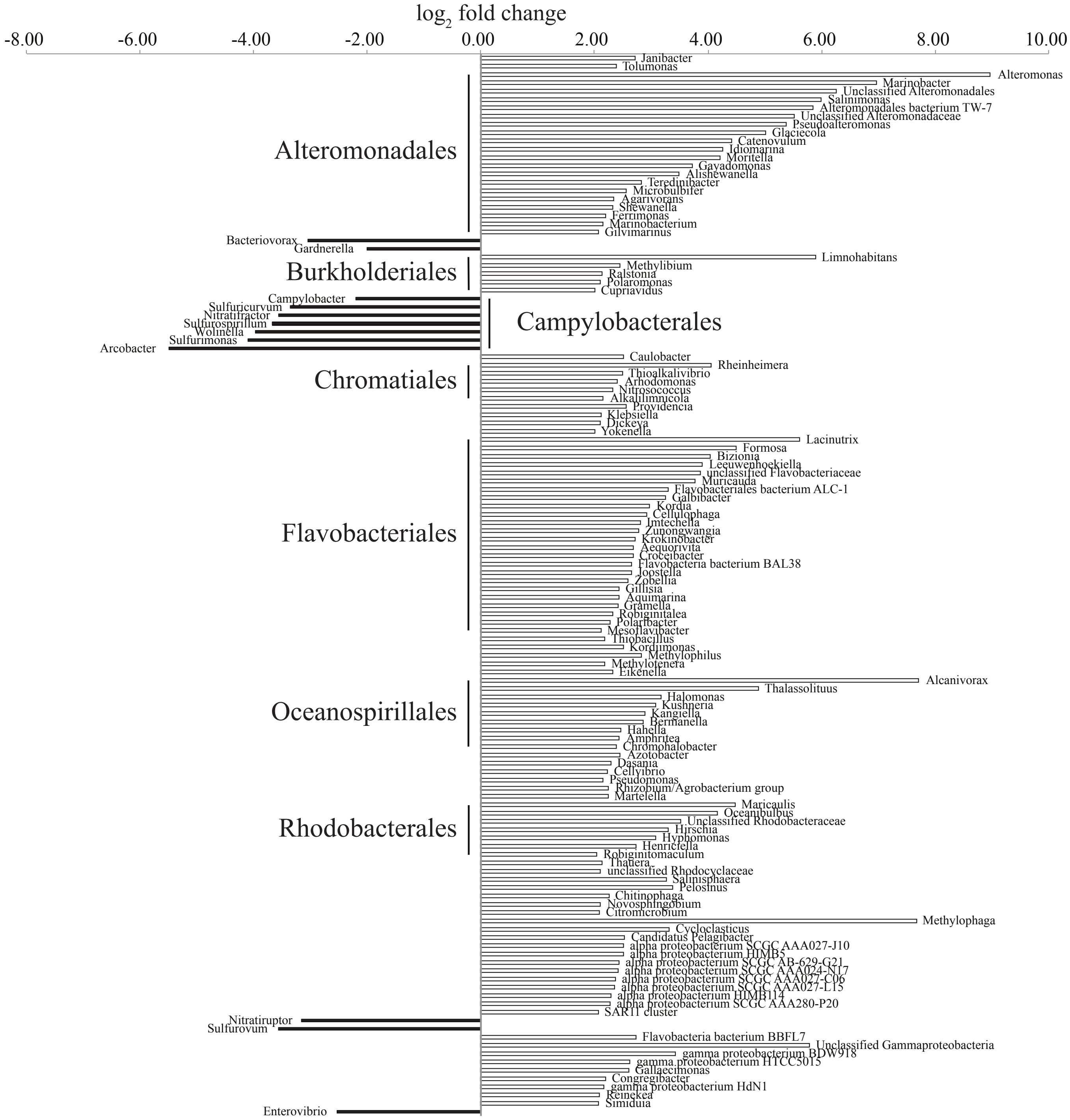

Figure A4. Bacterial genera that are significantly enriched (FDR < 1% and at least 4 $\log_2$ fold change in abundance) in a comparison among live traps, poisoned traps and surrounding seawater, according to the taxonomic identification of protein-coding sequences. Generalized lifestyles for selected genera are indicated next to genus names. Taxa which have been found in DOM enrichment events including phytoplankton blooms and experimental microcosms are also indicated.

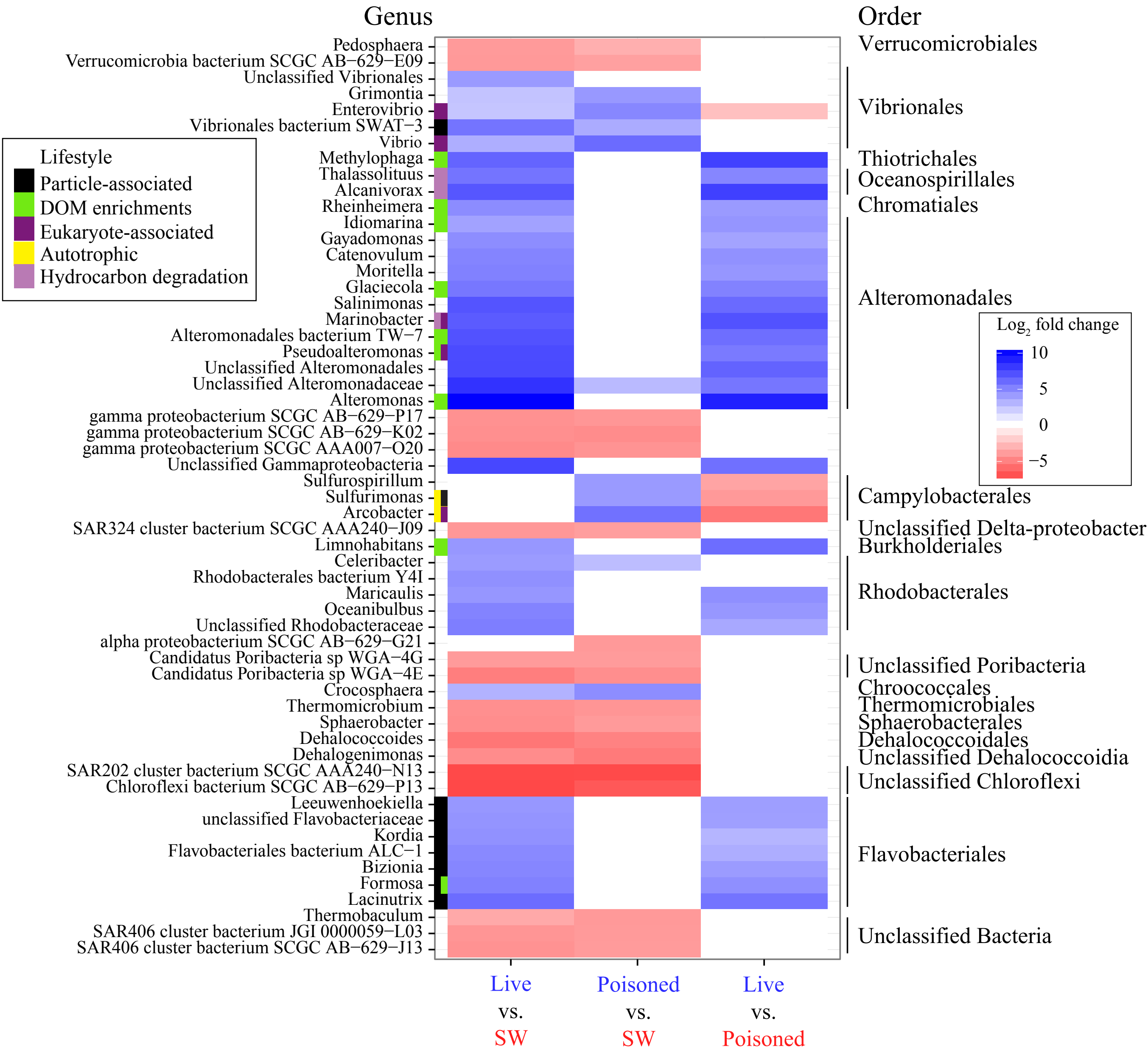

Figure A5. Abundance of eukaryotic OTUs, by phylum, relative to all sequences (including Bacteria and Archaea) in live (L, white) and poisoned (P, black) sediment traps as determined by the small subunit ribosomal RNA genes. The next taxonomic level above phylum is listed for taxa without phylum level designations in the SILVA database. Two or three-letter abbreviations for each taxon identification are listed along the top x-axis. Ap (Apicomplexa), As (Ascomycota), Ce (Cercozoa), Ci (Ciliophora), Dt (Diatomea), Di (Dinoflagellata), Eu (Euglenozoa), In (Incertae Sedis), Ja (Jakobida), La (Labyrinthulomycetes), Me (Metazoa), Pi Picozoa, Pr (Protalveolata), Re (Retaria), UA (Unclassified Alveolata), UCh (Unclassified Chloroplastida), UCr (Unclasified Crytophyceae), UE (Unclassified Eukaryota), UH (Unclassified Haptophyta), and US (Unclassified Stramenopiles).

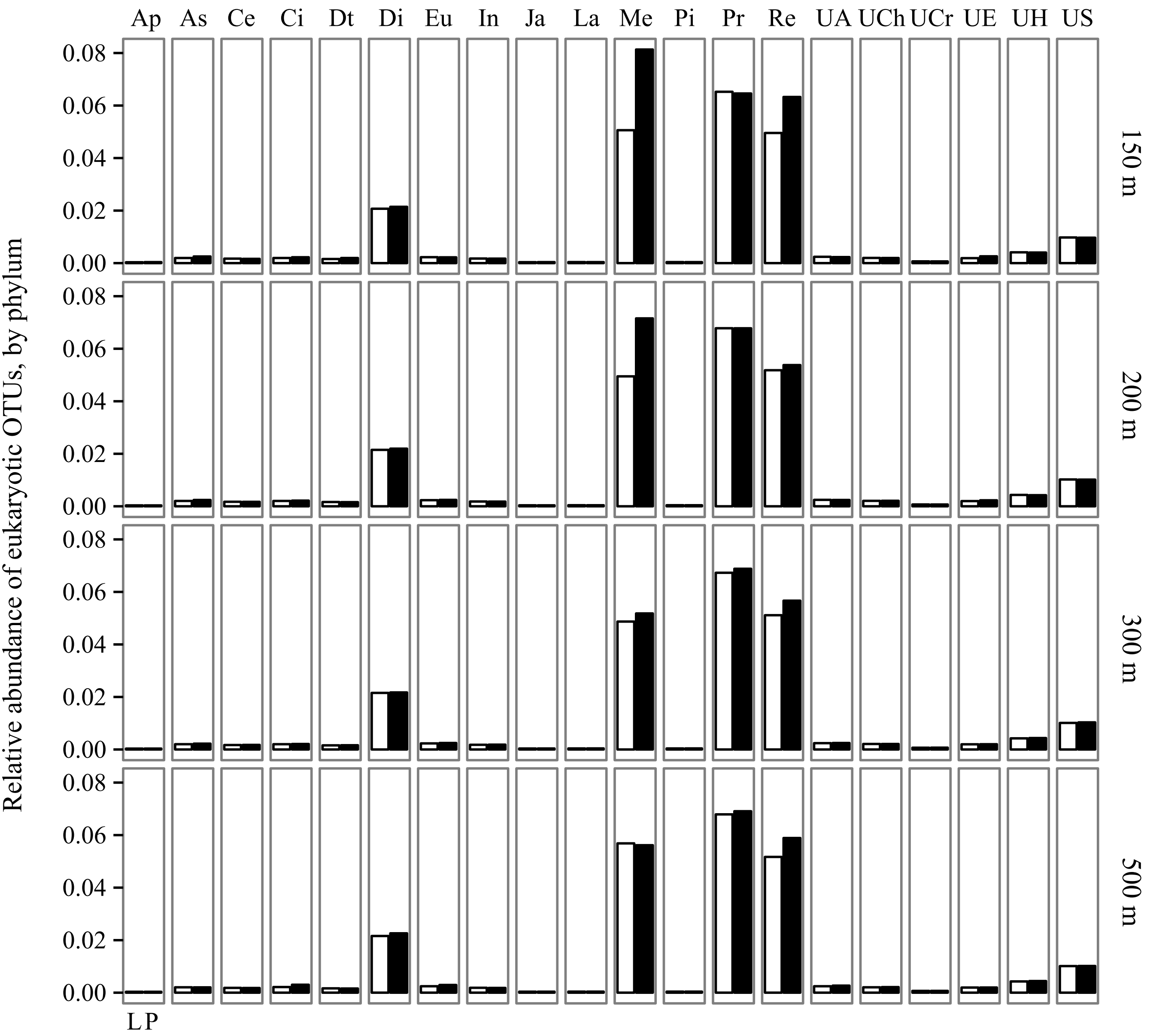

Figure A6. Microbial genera that are significantly enriched (FDR < 1%) in live (positive) or poisoned (negative) sediment traps, according to small subunit ribosomal RNA genes.

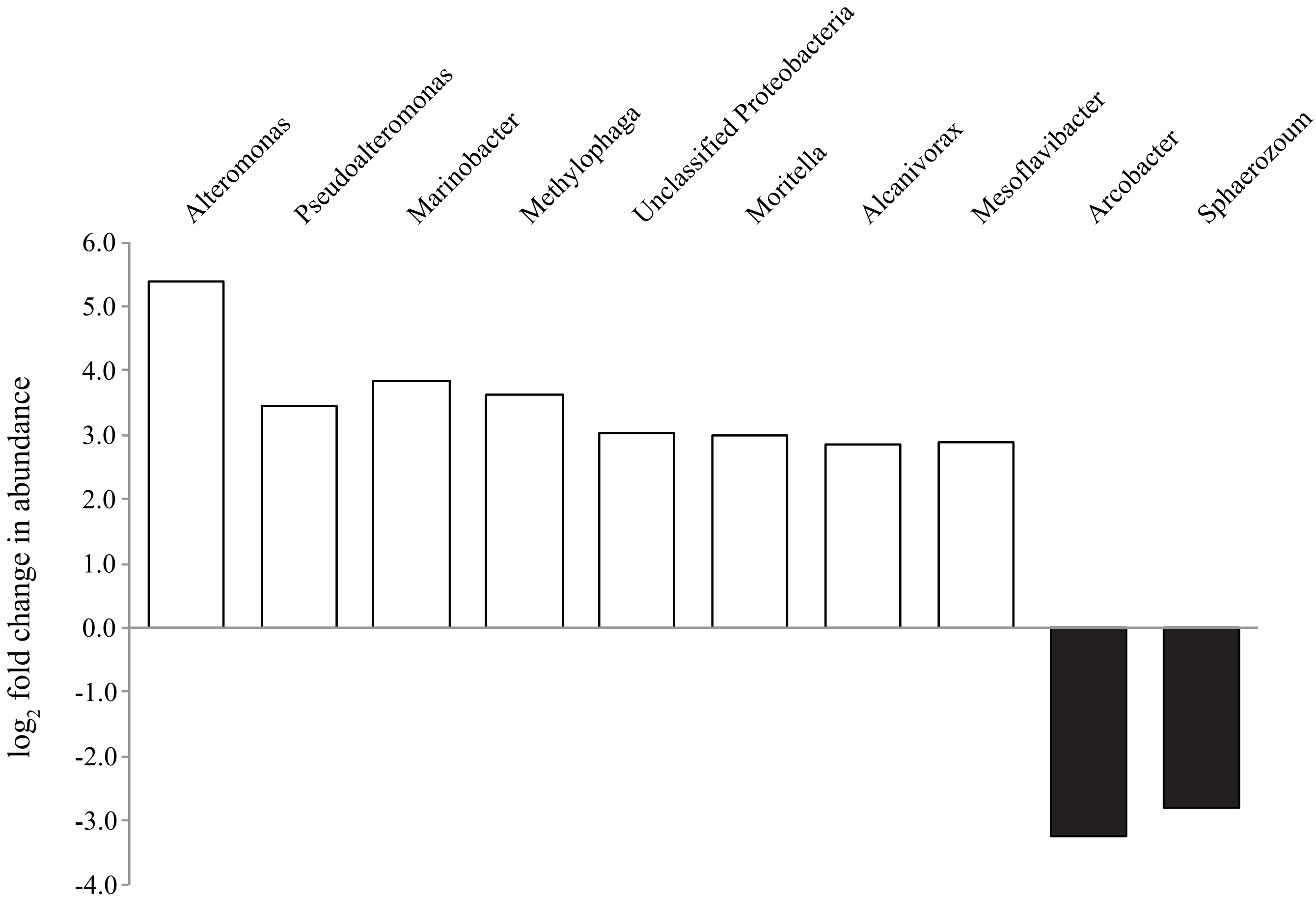

Figure A7. Functional pathways that are significantly enriched (FDR < 1%) in a comparison among live traps, poisoned traps and surrounding seawater, as classified by KEGG level 3. Generalized categories for selected pathways are indicated next to pathway names.

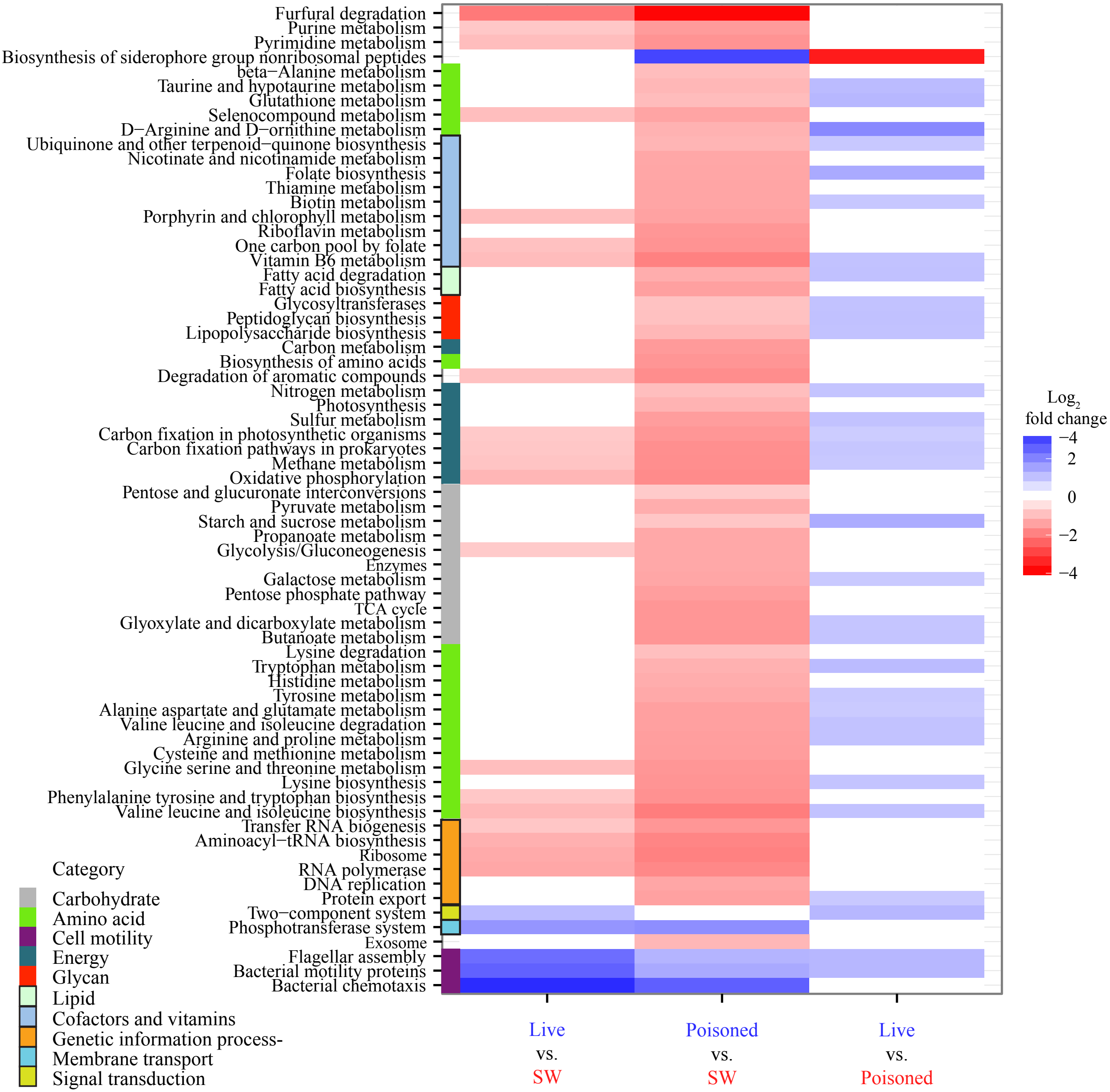

Figure A8. Enrichment of metal resistance genes on live marine particles . For full gene list see Dataset A2 in Appendix. (A) Selected metal resistance genes that are significantly enriched (FDR < 1%) in live as compared to poisoned sediment traps and (B) the most common genus of sequences matching the selected genes and its proportional representation within live (L) and poisoned (P) sediment traps at 150, 200, 300 and 500 meter depths. The first letter of each genus name is listed within each circle. The circle area represents the proportional representation of each genus within the specified sample. In cases where two genera each represented 50% of the sequences, both are listed.

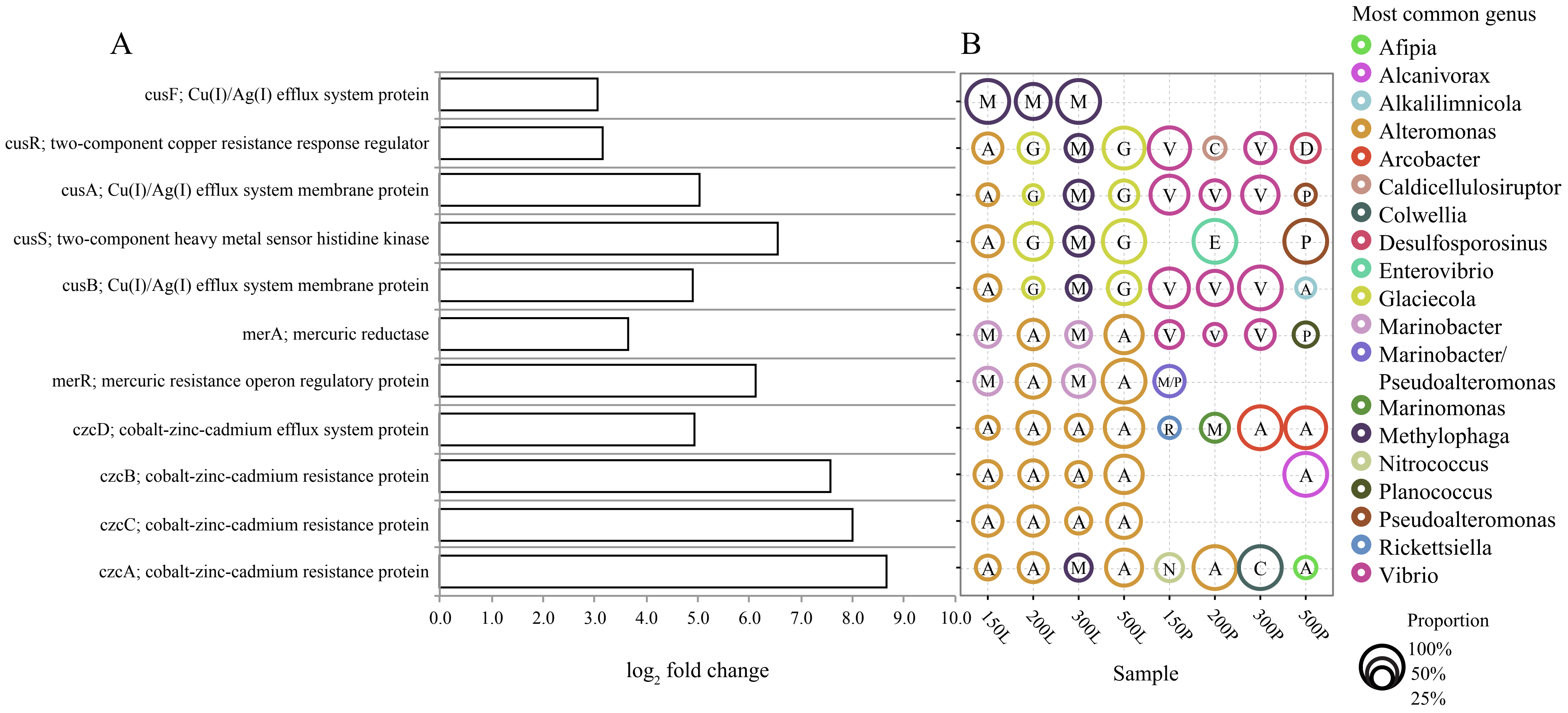

Supplement: Supplementary file 1 [file DataSheet1.PDF]
